# HRpI System Based on Wavenet Controller with Human Cooperative-in-the-Loop for Neurorehabilitation Purposes

**DOI:** 10.3390/s22207729

**Published:** 2022-10-12

**Authors:** Juan Daniel Ramirez-Zamora, Omar Arturo Dominguez-Ramirez, Luis Enrique Ramos-Velasco, Gabriel Sepulveda-Cervantes, Vicente Parra-Vega, Alejandro Jarillo-Silva, Eduardo Alejandro Escotto-Cordova

**Affiliations:** 1 Basic Sciences and Engineering Institute, Autonomous University of the State of Hidalgo—UAEH, Mineral de la Reforma 42184, Hidalgo, Mexico; 2 Aeronautical Engineering Department, Metropolitan Polytechnic University of Hidalgo—UPMH, Tolcayuca 43860, Hidalgo, Mexico; 3 Center for Innovation and Technological Development in Computing, National Polytechnic Institute—CIDETEC-IPN, Mexico City 07700, Mexico; 4 Robotics and Advanced Manufacturing Department, Research Center for Advanced Studies—CINVESTAV Saltillo, Ramos Arizpe 25900, Coahuila, Mexico; 5 Institute of Informatics, University of the South Sierra—UNSIS, Miahuatlán de Porfirio Díaz 70800, Oaxaca, Mexico; 6 Faculty of Higher Studies Zaragoza, National Autonomous University of Mexico—UNAM, Mexico City 09230, Mexico

**Keywords:** human robot physical interaction, artificial intelligence, assisting robotics

## Abstract

There exist several methods aimed at human–robot physical interaction (HRpI) to provide physical therapy in patients. The use of haptics has become an option to display forces along a given path so as to it guides the physiotherapist protocol. Critical in this regard is the motion control for haptic guidance to convey the specifications of the clinical protocol. Given the inherent patient variability, a conclusive demand of these HRpI methods is the need to modify online its response with neither rejecting nor neglecting interaction forces but to process them as patient interaction. In this paper, considering the nonlinear dynamics of the robot interacting bilaterally with a patient, we propose a novel adaptive control to guarantee stable haptic guidance by processing the causality of patient interaction forces, despite unknown robot dynamics and uncertainties. The controller implements radial basis neural network with daughter RASP1 wavelets activation function to identify the coupled interaction dynamics. For an efficient online implementation, an output infinite impulse response filter prunes negligible signals and nodes to deal with overparametrization. This contributes to adapt online the feedback gains of a globally stable discrete PID regulator to yield stiffness control, so the user is guided within a perceptual force field. Effectiveness of the proposed method is verified in real-time bimanual human-in-the-loop experiments.

## 1. Introduction

### 1.1. Background and Motivation

Research on motor rehabilitation therapy has provided advanced strategies for upper limb of people with cerebrovascular accident (CVA) [1,2], ranging from induced movement therapy [3], electromechanical assisted training [4], to robot-based haptics [5,6]. The emerging technologies of virtual reality [7,8], features rehabilitation by promoting repetition, and task-oriented training in a ludic, motivating and playful environment [9], facilitating functional, useful and improved experience. These not only benefit the user from this experience, but also the therapists who perform and evaluate and document online the tasks, with studies on upper limb motor recovery of CVA patient [10,11]. However, virtual body representation remains an involved issue [12], including its critical variable of interaction force of the patient [13], for viable and feasible interaction given the motor variability of each patient [14]. Platforms that integrate visual rendering with haptic stimuli conveys the user a multimodal perception for improved interaction [15]. There has been studied for motor training and/or rehabilitation protocols to generate a novel environment by executing the time and spatial patters of movements accurately through practice [16,17,18]. However, it remains unclear how to deal with volitional patient interaction force [19].

Neuropsychological rehabilitation offers also promising results based on programming specific tasks from the qualitative analysis of symptoms, some based on motor learning, i.e., changes in patient movements that reflect changes in the structure and function of the nervous system. In [20], it is presented an experimental study to improve the prediction time and reduce the robot response taken to reach a desired position, based on hand and eye-gaze motion determination, to foresee the point of user interaction in a virtual environment. However, the essential patient force interaction is not assessed. The development of haptic technology is incipient for rehabilitation purposes, though mature for computer and gaming interaction [21], it stands for subject of research how to deal patient interaction force, given their deviated motor patters, to promote their improvement under kinesthetic stimulus. It stands for a subject of research in several fields, including clinical tests have been implemented [22].

The so-called Porteus Maze Test (PMT) consists of solving mazes ordered in a pattern of increasing difficulty, it has been studied for upper limb rehabilitation of stroke patients; though PMT was proposed originally as a psychometric nonverbal test to measure psychological planning capacity and foresight (performance intelligence) [17]. The PMT is also currently used as a test associated with the activation of the frontal region of the brain, involved in the planning factor and capable of detecting perseverative errors [17]. Thus, it can be extended to evaluate executive functioning since it qualifies observable behaviors in neuropsychological rehabilitation [23], by inferring dysfunction of the Central Nervous System [24]. Then, it becomes a subject of interest to evaluate PMT under haptic guidance.

### 1.2. Contribution

A self-tuning scheme based on neural network identification of nonlinear dynamics is presented to adapt control feedback gains of a discrete PID guidance controller. Since a PID structure can be abstracted as the addition of restitution viscoelastic and memory generalized vector forces computed to converge to the nominal cartesian task, then the robot end-effector guides human user hand with such a vector force, which increases (diminishes) if position error increases (diminishes). Real-time experiments with nine healthy volunteers are presented under bilateral PMT using two Omni haptic interfaces. The user solves the virtual maze navigating under haptic guidance of the Omni at each hand under two modalities: (i) passive haptic guidance (PHG), where the user perceives a contact force each time he/she touches the maze boundaries, the less touches the better; (ii) active haptic guidance (AHG), where the user is guided continuously with a haptic force corresponding to how much it deviates from the nominal maze trajectory, the less position error the better. Results shows that in AHG renders improved trajectory precision from the self-tuning adaptation of force feedback.

## 2. The Problem Formulation

### 2.1. The Dynamics of the HRpI System

Consider a human–robot physical interaction system (HRpI) equipped with one haptic robotic device, one per left and right hand, that exhibits a high-end electromechanical performance, such as low friction, backdrivability and low inertia, with a high bandwidth to display force, whose nonlinear dynamic model is [25,26,27]

(1) $$H_{\sigma }\left(q_{\sigma }\right){\ddot{q}}_{\sigma }+C_{\sigma }\left(q_{\sigma },{\dot{q}}_{\sigma }\right){\dot{q}}_{\sigma }+G_{\sigma }\left(q_{\sigma }\right)+\tau_{f_{\sigma }}=\tau_{\sigma },$$

where σ is used to indicate left *l*, or right *r* haptic device, $q_{\sigma }\in R^{n},{\dot{q}}_{\sigma }\in R^{n}$ are the generalized position and velocity joint coordinates, respectively; $H_{\sigma }\left(q_{\sigma }\right)\in R^{n\times n}$ denotes a symmetric positive definite inertial matrix, $C_{\sigma }\left(q_{\sigma },{\dot{q}}_{\sigma }\right)\in R^{n\times n}$ represents the Coriolis and centripetal forces, $G_{\sigma }\left(q_{\sigma }\right)\in R^{n}$ models the gravity loads from earth gravitation field, and $\tau_{\sigma }\in R^{n}$ stands for the torque input. Term $\tau_{f_{\sigma }}=f_{b_{\sigma }}{\dot{q}}_{\sigma }+f_{c_{\sigma }}\tanh\left(\gamma_{\sigma }{\dot{q}}_{\sigma }\right)$ stands for joint friction, where $f_{b_{\sigma }},f_{c_{\sigma }}$ are positive definite n×n matrices modelling viscous damping and the dry friction respectively and its coefficient $\gamma_{\sigma }>0$. When the human operator is grasping the haptic device through placing its fingertip into its thimble, the dynamics changes remarkably due to human exerts a human torque $\tau_{h_{\sigma }}$ into the haptic robotic device:

(2) $$H_{\sigma }\left(q_{\sigma }\right){\ddot{q}}_{\sigma }+C_{\sigma }\left(q_{\sigma },{\dot{q}}_{\sigma }\right){\dot{q}}_{\sigma }+G_{\sigma }\left(q_{\sigma }\right)+\tau_{f_{\sigma }}=\tau_{\sigma }+\tau_{h_{\sigma }},$$

where $\tau_{h_{\sigma }}$ is assumed Liptchitz, giving rise to a human-in-the-loop configuration [26]. System (2) can be written in continuous time state space representation $x_{\sigma }={\left[x_{\sigma_{1}}x_{\sigma_{2}}\right]}^{T}={\left[q_{\sigma }{\dot{q}}_{\sigma }\right]}^{T}$ as follows

(3) $$\begin{array}{ccc} {\dot{x}}_{\sigma }\left(t\right) & = & f_{\sigma }\left(x_{\sigma }\left(t\right)\right)+g_{\sigma }\left(x_{\sigma }\left(t\right)\right)u_{\sigma }\left(t\right)+g_{\sigma }\left(x_{\sigma }\left(t\right)\right)\tau_{h_{\sigma }}\left(t\right) \end{array}$$

(4) $$\begin{array}{ccc} y_{\sigma }\left(t\right) & = & c_{\sigma }x_{\sigma }\left(t\right) \end{array}$$

where $g_{\sigma }\left(x_{\sigma }\left(t\right)\right)\tau_{h_{\sigma }}\left(t\right)$ is the map of the exogenous time-varying unmeasurable human torque, and

(5) $$f_{\sigma }\left(x_{\sigma }\left(t\right)\right)=\left[\begin{array}{c} x_{\sigma_{2}}\\ -H_{\sigma }^{-1}\left[C_{\sigma }x_{\sigma_{2}}+G_{\sigma }+\tau_{f_{\sigma }}\right] \end{array}\right],g_{\sigma }\left(x_{\sigma }\left(t\right)\right)=\left[\begin{array}{c} 0\\ H_{\sigma }^{-1} \end{array}\right]$$

are unknown smooth functions, and $u_{\sigma }=\tau_{\sigma }$ is the control input.

### 2.2. Problem Statement

**Figure 1:**
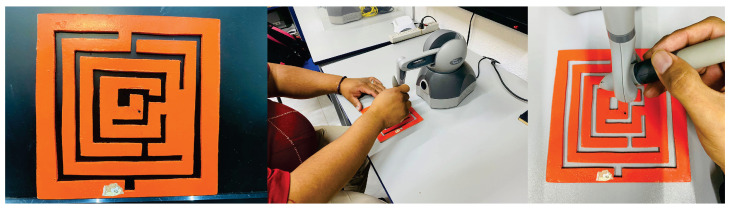
Patients under haptic guidance carrying out a maze task. These studies suggest that adaptability of the control strategy promotes rehabilitation evidence of motor patterns.

When the human operator has a motor disability, there has been proposed non-linear robot controllers $u_{\sigma }$ to assist motion [28,29,30], including a high performance decentralized continuous nonlinear PID controller [31], however with constant feedback gains that do not adapt to changing conditions, such a time varying persistent human interaction term $g_{\sigma }\left(x_{\sigma }\left(t\right)\right)\tau_{h_{\sigma }}\left(t\right)$. Then, the problem can be stated as follows: assuming unknown $f_{\sigma }\left(x_{\sigma }\left(t\right)\right)$ and unknown human interaction generalized force $g_{\sigma }\left(x_{\sigma }\left(t\right)\right)\tau_{h_{\sigma }}\left(t\right)$, design a model-free control $u_{\sigma }$ with feedback gains that adapts online for each patient so as to his/her performance improves when interacting with the robotic device under haptic guidance. Figure 1 shows a maze solution application.

## 3. Adaptive Interaction System

**Figure 2:**
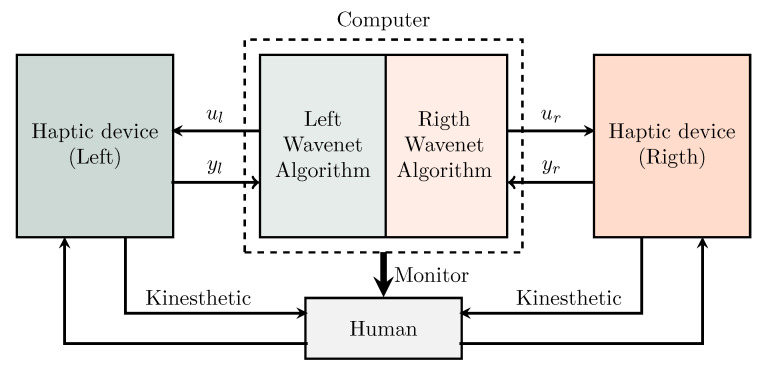
Block diagram of the bimanual cooperative system, showing the multimodal stimuli to the human, consequently the human decision making process solves cooperativeness to issue simultaneously motor commands to each device.

In this section, the adaptive interactive system is presented as shown in Figure 2, as can be seen each of the haptic device has a programmed wavenet PID controller which has communication between them through the computer where the algorithms are run. The wavenet PID controller scheme is based on an identification of inverse dynamics of each haptic device and a IIR filter to tune PID feedback gains and guarantees global regulation.

**Figure 3:**
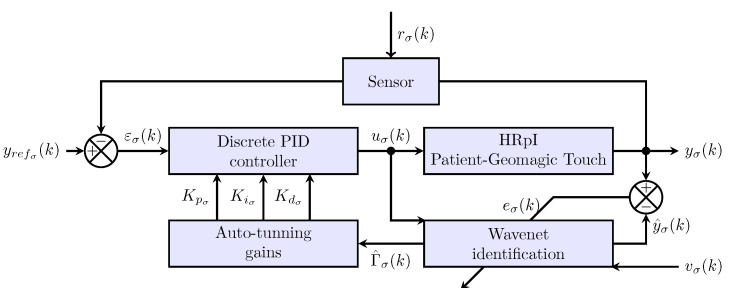
PID Wavenet controller scheme where σ can be left, *l* or right, *r*, i.e., σ={l,r}, $y_{ref_{\sigma }}\left(k\right)$ is the reference signal, $\varepsilon_{\sigma }\left(k\right)$ stands for the error signal, the control input is $u_{\sigma }\left(k\right)$, $r_{\sigma }\left(k\right)$ models the noise signal, $y_{\sigma }\left(k\right)$ is the HRpI (human patient in the haptic loop ) output with ${\hat{y}}_{\sigma }\left(k\right)$ its estimate, and $e_{\sigma }\left(k\right)$ the error estimated, finally, $v_{\sigma }\left(k\right)$ stands for the persistence signal.

For this purpose, the control scheme shown in Figure 3 is used, where four main blocks can be observed: HRpI, Wavenet identification, Discrete PID controller and Auto-tunning gains. The following subsections will describe each of them.

### 3.1. Input-Output Dynamics of the HRpI

It is well-known that any sufficiently smooth continuous time non-linear system admits a discrete-time representation [32,33]. Then, (2) or (3),(4) can be represented in discrete time state space, by assuming access to all state at each time, with small enough sampling period T>0, and provided that input remains constant between sampling instant $I_{k}=\left[kT\left(k+1\right)T\right]$, where k≥0. In this way, (3) is approximated with a first order Euler forward difference ${\dot{x}}_{\sigma }\approx \frac{x_{\sigma }\left(t+T\right)-x_{\sigma }\left(t\right)}{T}$ as follows

(6) $$\frac{x_{\sigma }\left(t+T\right)-x_{\sigma }\left(t\right)}{T}=f_{\sigma }\left(x_{\sigma }\left(t\right)\right)+g_{\sigma }\left(x_{\sigma }\left(t\right)\right)u_{\sigma }\left(t\right).$$

Solving (6) for $x_{\sigma }\left(t+T\right)$ leads to

(7) $$x_{\sigma }\left(t+T\right)=x_{\sigma }\left(t\right)+f_{\sigma }\left(x_{\sigma }\left(t\right)\right)T+g_{\sigma }\left(x_{\sigma }\left(t\right)\right)Tu_{\sigma }\left(t\right).$$

Evaluating (7) and (4) at t=kT yields the following nonlinear discrete time system

(8) $$\begin{array}{cc} x_{\sigma }\left[\left(k+1\right)T\right]= & x_{\sigma }\left[kT\right]+f_{\sigma }\left(x_{\sigma }\left[kT\right]\right)T+g_{\sigma }\left(x_{\sigma }\left[kT\right]\right)Tu_{\sigma }\left[kT\right] \end{array}$$

(9) $$\begin{array}{cc} y_{\sigma }\left[\left(k+1\right)T\right]= & c_{\sigma }x_{\sigma }\left[\left(k+1\right)T\right]. \end{array}$$

Substituting (8) into (9), the discrete output at instant (k+1)T is given by

(10) $$\begin{array}{ccc} y_{\sigma }\left[\left(k+1\right)T\right]\; & = & C_{\sigma }\left(x_{\sigma }\left[kT\right]+f_{\sigma }\left(x_{\sigma }\left[kT\right]\right)T\right)+C_{\sigma }\left(g_{\sigma }\left(x_{\sigma }\left[kT\right]\right)T\right)u_{\sigma }\left[kT\right]\\ & ≜ & \Phi_{\sigma }\left[x_{\sigma }\left[kT\right],T\right]+\Gamma_{\sigma }\left[x_{\sigma }\left[kT\right],T\right]u_{\sigma }\left[kT\right] \end{array}$$

where $\Phi_{\sigma }\left(*\right)$ stands for the flow of discrete dynamic system with $\Gamma_{\sigma }\left(*\right)$ the input matrices of robot, with

(11) $$\begin{array}{ccc} \Phi_{\sigma }\left[x_{\sigma }\left[kT\right],T\right] & = & c_{\sigma }\left(x_{\sigma }\left[kT\right]+f_{\sigma }\left(x_{\sigma }\left[kT\right]\right)T\right) \end{array}$$

(12) $$\begin{array}{ccc} \Gamma_{\sigma }\left[x_{\sigma }\left[kT\right],T\right] & = & c_{\sigma }\left(g_{\sigma }\left(x_{\sigma }\left[kT\right]\right)T\right). \end{array}$$

Notice that (10) describes the input-output dynamics of the haptic device σ={l,r}, at instant k+1. Notice that input $u_{\sigma }\left(k\right)$ and system output $y_{\sigma }\left(k\right)$ are the only data available. In this paper, we exploit the properties of wavenets to approximate the input-output dynamics (10) of each haptic device, but additionally we consider IIR filter in the output layer to prune irrelevant signals to build an efficient identification scheme useful to tune PID feedback gains [34].

### 3.2. Wavenet Identification

**Figure 4:**
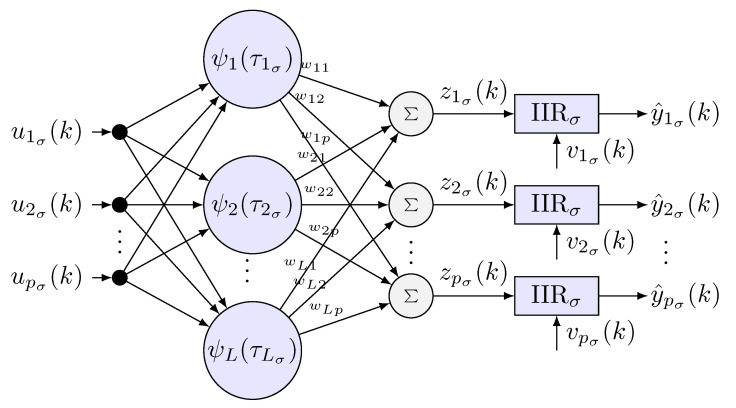
Diagram of a wavenet neural network with an IIR filter in cascade where σ can be left, *l* or right, *r*, i.e., σ={l,r}.

A scheme is proposed to identify approximately the inverse plant (HRpI system), to this end, a radial basis neural network is used. The activation functions $\psi (\tau_{\sigma })$ are daughter wavelet functions $\psi_{j}\left(\tau_{\sigma }\right)$ of RASP1 type. To filter neurons that have little contribution in the identification process, three IIR filters are incorporated in cascade, using the least number of neurons possible and reduce the computational load in the learning process [35]. In Figure 4, the signal propagation and the general interconnection are presented, where

$$\begin{array}{c} \tau_{L_{\sigma }}=\frac{∥u_{\sigma }\left(k\right)-b_{L_{\sigma }}∥}{a_{L_{\sigma }}}. \end{array}$$

**Figure 5:**
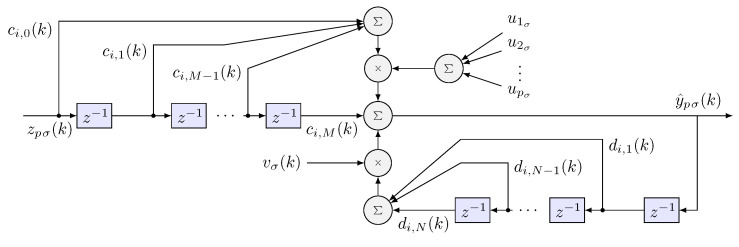
IIR filter structure.

Infinite impulse response (IIR) recurrent structure (Figure 5), in cascading structure, yields double improving speed of learning by pruning those nodes with insignificant relevant information from the cross contribution summation of daugthers wavelets. Notice in the scheme, the forward delayed structure modulated by the input and the feedback loop modulated by the persistent signal to allows swapping a range of frequency [36].

The mother wavelet function ψ(k) generates daughter wavelets $\psi_{L}\left(\tau_{L\sigma }\right)$ by its property of expansion or contraction and translation, represented as [37]:

(13) $$\psi_{L}\left(\tau_{L_{\sigma }}\right)=\frac{1}{\sqrt{a}}\psi \left(\tau_{L_{\sigma }}\right)$$

with a≠0; a,b∈R and

(14) $$\tau_{L_{\sigma }}={\left(\begin{array}{c} \sum_{j=1}^{p}{\left(u_{j}-b_{L_{\sigma }}\right)}^{2} \end{array}\right)}^{1/2}/a_{L_{\sigma }}$$

the *j* scale variable, $a_{L_{\sigma }}$ allows expansion and contraction, and $b_{L_{\sigma }}$ stands for the $\left(L_{\sigma }\right)$ translation variable at *k*, in the classical role of RBF, with the advantage of dealing with more refinement through daughters wavelets $\psi_{L}\left(\tau_{L_{\sigma }}\right)$. This last feature is essential in the present algorithm together with the pruning capability of the IIR filter. As suggested in [37], the mathematical representation of wavelet RASP1 is a singularity-free normalization of the argument of the wavelet

(15) $$RASP1_{\sigma }=\frac{\tau_{\sigma }}{{\left(\tau_{\sigma }^{2}+1\right)}^{2}}$$

whose partial derivative with respect to $b_{L_{\sigma }}$ is

(16) $$\frac{\partial RASP1_{\sigma }}{\partial b_{L_{\sigma }}}=\frac{1}{a}\frac{3\tau_{\sigma }^{2}-1}{{\left(\tau_{\sigma }^{2}+1\right)}^{3}}$$

In this way, for the letf o rigth haptic device, the *i* wavenet approximation signal with IIR filter can be calculated as:

(17) $$\begin{array}{ccc} {\hat{y}}_{i_{\sigma }}\left(k\right) & = & \sum_{q=1}^{p}\sum_{l=0}^{M}c_{i,l}z_{i_{\sigma }}\left(k-l\right)u_{q_{\sigma }}\left(k\right)+\sum_{j=1}^{N}d_{i,j}{\hat{y}}_{i_{\sigma }}\left(k-j\right)v_{\sigma }\left(k\right) \end{array}$$

(18) $$\begin{array}{ccc} z_{i_{\sigma }}\left(k\right) & = & \sum_{l=1}^{L}w_{i,l}\psi_{l_{\sigma }}\left(k\right) \end{array}$$

where *L* stands for the number of daughter wavelets, $w_{i,l}$ the weights of each neuron in the wavenet, $c_{i,l}$ and $d_{i,j}$ are the coefficients of forward and backward IIR filter, respectively, and *M* and *N* the coefficients number of forward and backward IIR${}_{\sigma }$ filter, respectively. As can be seen, the (17) has the following matrix structure

(19) $${\hat{y}}_{\sigma }\left(k+1\right)={\hat{\Phi }}_{\sigma }\left[y_{\sigma }\left(k\right),\Theta_{\Phi_{\sigma }}\right]+{\hat{\Gamma }}_{\sigma }\left[y_{\sigma }\left(k\right),\Theta_{\Gamma_{\sigma }}\right]\cdot u_{\sigma }\left(k\right).$$

System (19) is estimated by two wavenet functions as follows

(20) $$\begin{array}{ccc} {\hat{\Phi }}_{\sigma }\left[y_{\sigma }\left(k\right),\Theta_{\Phi_{\sigma }}\right] & = & \sum_{j=1}^{N}d_{i,j}{\hat{y}}_{i_{\sigma }}\left(k-j\right)v_{\sigma }\left(k\right) \end{array}$$

(21) $$\begin{array}{ccc} {\hat{\Gamma }}_{\sigma }\left[y_{\sigma }\left(k\right),\Theta_{\Gamma_{\sigma }}\right] & = & \sum_{l=0}^{M}c_{i,l}z_{i_{\sigma }}\left(k-l\right) \end{array}$$

with adjustable parameters $\Theta_{\Phi_{\sigma }}$ and $\Theta_{\Gamma_{\sigma }}$. Therefore, since nonlinear functions wavenet functions ${\hat{\Phi }}_{\sigma }\left(k\right)$ and ${\hat{\Gamma }}_{\sigma }\left(k\right)$ estimate $\Phi_{\sigma }\left(k\right)$ and $\Gamma_{\sigma }\left(k\right)$, as k→∞, then error $e_{i_{\sigma }}\left(k\right)=y_{i_{\sigma }}\left(k\right)-{\hat{y}}_{i_{\sigma }}\left(k\right)\to 0$ can be used as a Lebesgue measure useful to tune feedback gains.

#### Weavenet Learning

The parameters of the neural network and the IIR filters in their matrix form are: control signal $u_{\sigma }={\left[u_{1_{\sigma }},\;u_{2_{\sigma }},\ldots ,u_{p_{\sigma }}\right]}^{T}$, the translation parameter $b_{\sigma }={\left[b_{1_{\sigma }},\;b_{2_{\sigma }},\ldots ,b_{L_{\sigma }}\right]}^{T}$, the dilatation parameter $a_{\sigma }={\left[a_{1_{\sigma }},\;a_{2_{\sigma }},\ldots ,a_{L_{\sigma }}\right]}^{T}$, the daughter wavelets $\psi_{\sigma }={\left[\psi_{1},\psi_{2},\ldots ,\psi_{L}\right]}^{T}$, the neural network output $z_{\sigma }={\left[z_{1_{\sigma }},\;z_{2_{\sigma }},\;\ldots ,\;z_{p_{\sigma }}\right]}^{T}$, the estimated position ${\hat{y}}_{\sigma }=[{\hat{y}}_{1_{\sigma }},\;{\hat{y}}_{2_{\sigma }},$$\;\ldots ,\;{\hat{y}}_{p_{\sigma }}{]}^{T}$, and the synaptic weight matrices $W_{\sigma }\in R^{p\times L}$; and the coefficients $C_{\sigma }\in R^{p\times M}$ and $D_{\sigma }\in R^{p\times N}$ for the filters are:

(22) $$\begin{array}{ccc} W_{\sigma } & = & \left[\begin{array}{cccc} w_{11} & w_{12} & \cdots & w_{1p}\\ w_{21} & w_{22} & \cdots & w_{2p}\\ \vdots & \vdots & \ddots & \vdots \\ w_{p1} & w_{p2} & \cdots & w_{Lp} \end{array}\right],C_{\sigma }=\left[\begin{array}{cccc} c_{10} & c_{11} & \cdots & c_{1M}\\ c_{20} & c_{21} & \cdots & c_{2M}\\ \vdots & \vdots & \ddots & \vdots \\ c_{p1} & c_{p2} & \cdots & c_{pM} \end{array}\right],\\ D_{\sigma } & = & \left[\begin{array}{cccc} d_{11} & d_{12} & \cdots & d_{1N}\\ d_{21} & d_{22} & \cdots & d_{2N}\\ \vdots & \vdots & \ddots & \vdots \\ d_{p1} & d_{p2} & \cdots & d_{pN} \end{array}\right]. \end{array}$$

The output $z_{\sigma }\left(k\right)$ of the wavenet is given by

(23) $$\begin{array}{cc} z_{\sigma }\left(k\right) & =u_{\sigma }^{T}\left(k\right)W_{\sigma }\left(k\right)\psi_{\sigma }^{T}\left(k\right), \end{array}$$

which is passed through the IIR filters to obtain the estimated position ${\hat{y}}_{\sigma }\left(k\right)$,

(24) $${\hat{y}}_{\sigma }\left(k\right)={\hat{\Gamma }}_{\sigma }\left(k\right)+D_{\sigma }{\hat{Y}}_{\sigma }\left(k\right)v_{\sigma }\left(k\right)$$

where

(25) $$\begin{array}{cc} {\hat{\Gamma }}_{\sigma }\left(k\right) & =C_{\sigma }z_{\sigma }\left(k\right), \end{array}$$

(26) $$\begin{array}{cc} {\hat{Y}}_{\sigma }\left(k\right) & =\left[\begin{array}{cccc} {\hat{y}}_{1}\left(k-1\right) & {\hat{y}}_{1}\left(k-2\right) & \cdots & {\hat{y}}_{1}\left(k-N\right)\\ {\hat{y}}_{2}\left(k-1\right) & {\hat{y}}_{2}\left(k-2\right) & \cdots & {\hat{y}}_{2}\left(k-N\right)\\ \vdots & \vdots & \ddots & \vdots \\ {\hat{y}}_{p}\left(k-1\right) & {\hat{y}}_{p}\left(k-2\right) & \cdots & {\hat{y}}_{p}\left(k-N\right) \end{array}\right] \end{array}$$

and $v_{\sigma }\left(k\right)={\left[\begin{array}{cccc} v_{1_{\sigma }\left(k\right)} & v_{p_{\sigma }\left(k\right)} & \ldots & v_{p_{\sigma }} \end{array}\right]}^{T}$ is the persistent filter signal.

The wavenet parameters are optimized by a least mean square algorithm (LMS) subject to minimizing a convex radially unbounded cost functions $E_{\sigma }$, defined by

(27) $$E_{\sigma }\left(k\right)=\frac{1}{2}\sum_{i=1}^{p}{\left[e_{i_{\sigma }}\left(k\right)\right]}^{2}.$$

Let the estimation error between wavenet output signal with IIR${}_{\sigma }$ filter and system output be

(28) $$e_{i_{\sigma }}\left(k\right)=y_{i_{\sigma }}\left(k\right)-{\hat{y}}_{i_{\sigma }}\left(k\right).$$

To minimize $E_{\sigma }\left(k\right)$, the steepest gradient-descent method is considered. To this end, notice that partial derivatives of $E_{\sigma }\left(k\right)$ with respect to $a_{\sigma }\left(k\right)$, $b_{\sigma }\left(k\right)$, $W_{\sigma }\left(k\right)$, $C_{\sigma }\left(k\right)$ and $D_{\sigma }\left(k\right)$ are required for each haptic device to update the incremental changes of each parameter along its negative gradient direction. That is,

(29) $$\begin{array}{ccc} \Delta W_{\sigma }\left(k\right) & = & -\frac{\partial E_{\sigma }\left(k\right)}{\partial W_{\sigma }\left(k\right)} \end{array}$$

(30) $$\begin{array}{ccc} \Delta a_{\sigma }\left(k\right) & = & -\frac{\partial E_{\sigma }\left(k\right)}{\partial a_{\sigma }\left(k\right)} \end{array}$$

(31) $$\begin{array}{ccc} \Delta b_{\sigma }\left(k\right) & = & -\frac{\partial E_{\sigma }\left(k\right)}{\partial b_{\sigma }\left(k\right)} \end{array}$$

(32) $$\begin{array}{ccc} \Delta C_{\sigma }\left(k\right) & = & -\frac{\partial E_{\sigma }\left(k\right)}{\partial C_{\sigma }\left(k\right)} \end{array}$$

(33) $$\begin{array}{ccc} \Delta D_{\sigma }\left(k\right) & = & -\frac{\partial E_{\sigma }\left(k\right)}{\partial D_{\sigma }\left(k\right)} \end{array}$$

then, the tuning update parameter for each haptic device becomes:

(34) $$\begin{array}{ccc} W_{\sigma }\left(k+1\right) & = & W_{\sigma }\left(k\right)+\mu_{W_{\sigma }}\Delta W_{\sigma }\left(k\right) \end{array}$$

(35) $$\begin{array}{ccc} a_{\sigma }\left(k+1\right) & = & a_{\sigma }\left(k\right)+\mu_{a_{\sigma }}\Delta a_{\sigma }\left(k\right) \end{array}$$

(36) $$\begin{array}{ccc} b_{\sigma }\left(k+1\right) & = & b_{\sigma }\left(k\right)+\mu_{b_{\sigma }}\Delta b_{\sigma }\left(k\right) \end{array}$$

(37) $$\begin{array}{ccc} C_{\sigma }\left(k+1\right) & = & C_{\sigma }\left(k\right)+\mu_{C_{\sigma }}\Delta C_{\sigma }\left(k\right) \end{array}$$

(38) $$\begin{array}{ccc} D_{\sigma }\left(k+1\right) & = & D_{\sigma }\left(k\right)+\mu_{D_{\sigma }}\Delta D_{\sigma }\left(k\right) \end{array}$$

### 3.3. Discrete PID Controller for Each Haptic Device

The following discrete PID controller is proposed:

(39) $$\begin{array}{ccc} u_{\sigma }\left(k+1\right) & = & u_{\sigma }\left(k\right)+k_{p_{\sigma }}\left(k\right)\left[\varepsilon_{\sigma }\left(k\right)-\varepsilon_{\sigma }\left(k-1\right)\right]+\\ & & k_{d_{\sigma }}\left(k\right)\left[\varepsilon_{\sigma }\left(k\right)-2\varepsilon_{\sigma }\left(k-1\right)+\varepsilon_{\sigma }\left(k-2\right)\right]+\\ & & k_{i_{\sigma }}\left(k\right)\varepsilon_{\sigma }\left(k\right) \end{array}$$

where $k_{p_{\sigma }}\left(k\right)$, $k_{i_{\sigma }}\left(k\right)$ and $k_{d_{\sigma }}\left(k\right)$ stand for strictly positive definite proportional, integral and derivative feedback gains, respectively; $u_{\sigma }\left(k\right)$ is the controller at instant *k*, and error is defined as

(40) $$\varepsilon_{\sigma }\left(k\right)=y_{ref_{\sigma }}\left(k\right)-y_{\sigma }\left(k\right)$$

for σ={l,r}. Each feedback gain is tuned according to the corresponding error they affect in (39) and modulated by ${\hat{\Gamma }}_{\sigma }$, the input matrix of (19).

### 3.4. Auto-Tuning PID Gains

Due to the gains $k_{p_{\sigma }}$, $k_{i_{\sigma }}$ and $k_{d_{\sigma }}$ are considered within the cost function (27), those can be updated similar to (34)–(38). Let

(41) $$\begin{array}{ccc} k_{p_{\sigma }}\left(k\right) & = & k_{p_{\sigma }}\left(k-1\right)+\mu_{p}e_{\sigma }\left(k\right){\hat{\Gamma }}_{i,q}\left(k\right)\left[\varepsilon_{\sigma }\left(k\right)-\varepsilon_{\sigma }\left(k-1\right)\right] \end{array}$$

(42) $$\begin{array}{ccc} k_{i_{\sigma }}\left(k\right) & = & k_{i_{\sigma }}\left(k-1\right)+\mu_{i}e_{\sigma }\left(k\right){\hat{\Gamma }}_{i,q}\left(k\right)\varepsilon_{\sigma }\left(k\right)\\ k_{d_{\sigma }}\left(k\right) & = & k_{d_{\sigma }}\left(k-1\right)+\mu_{d}e_{\sigma }\left(k\right){\hat{\Gamma }}_{i,q}\left(k\right) \end{array}$$

(43) $$\begin{array}{ccc} & & \left[\varepsilon_{\sigma }\left(k\right)-2\varepsilon_{\sigma }\left(k-1\right)+\varepsilon_{\sigma }\left(k-2\right)\right] \end{array}$$

where ${\hat{\Gamma }}_{\sigma }$ is defined by (21), for 0<μ<1 the learning rate of the PID controller gains. Notice that learning rates μ are designer parameters and are used for both controllers.

### 3.5. PID Wavenet Controller Algorithm

The proposed PID wavenet Algorithm 1 is summarized as follows:   


**Algorithm 1: **PID Wavenet Controller
1:**Read** the wavenet parameters: number of neurons $L_{\sigma }$, learning rates ($\mu_{W_{\sigma }},\;\mu_{a_{\sigma }},\;\mu_{b_{\sigma }}$), synaptic weight values $W_{\sigma }$ and the wavelet $\psi_{\sigma }$.2:**Read** the IIR filter parameters: number of feed-backs and feed-forward coefficients, $M_{\sigma }$ and $N_{\sigma }$, respectively; learning rates ($\mu_{C_{\sigma }},\;\mu_{D_{\sigma }}$), the IIR coefficient values ($C_{\sigma }\;D_{\sigma }$) and the persistent signal $v_{\sigma }\left(k\right)$.3:**Read** the PID controller parameters: gain values ($K_{p_{\sigma }},\;K_{i_{\sigma }},\;K_{d_{\sigma }}$) and its update rates ($\mu_{p_{\sigma }},\;\mu_{i_{\sigma }},\;\mu_{d_{\sigma }}$).4:**Read** the operation parameters: number of epochs epks.5:Initialize the internal parameters and k=0.6:**while** it is working **do**7:    **for** epk=0;epk≤epks;i++ **do**8:        **Get** the control signal $u_{\sigma }\left(k\right)$ and the output from plant $y_{\sigma }\left(k\right)$, Equations (39) and (9).9:        **Compute** the vector $\tau_{L_{\sigma }\left(k\right)}$ using (14).10:        Evaluate the mother’s wavelet $\psi_{L}\left(\tau_{L_{\sigma }}\left(k\right)\right)$ with (13).11:        **Compute** the wavelet output $z_{\sigma }\left(k\right)$ with (23).12:        **Compute** estimated ${\hat{y}}_{\sigma }\left(k\right)$ using (24).13:        **Compute** the estimated error using (28).14:        **Compute** the energy function $E_{\sigma }\left(k\right)$ with (27).15:        **Compute** the parameter gradients: $\Delta W_{\sigma }\left(k\right)$, $\Delta a_{\sigma }\left(k\right)$, $\Delta b_{\sigma }\left(k\right)$, $\Delta C_{\sigma }\left(k\right)$, $\Delta D_{\sigma }\left(k\right)$, Equations (29)–(33).16:        **Update** the parameter values using its learning ratios: $\Delta W_{\sigma }\left(k+1\right)$, $\Delta a_{\sigma }\left(k+1\right)$, $\Delta b_{\sigma }\left(k+1\right)$, $\Delta C_{\sigma }\left(k+1\right)$, $\Delta D_{\sigma }\left(k+1\right)$; Equations (34)–(38).17:    **end for**18:    Get the parameter ${\hat{\Gamma }}_{\sigma }\left(k\right)$ and the tracking error $\varepsilon_{\sigma }\left(k\right)$, Equations (25) and (40).19:    Tune controller gains: $K_{p_{\sigma }}\left(k\right),\;K_{i_{\sigma }}\left(k\right),\;K_{d_{\sigma }}\left(k\right)$: Equations (41)–(43).20:    Calculate the new control signal u(k+1) using (39).21:    Reassign the new values.22:    Increase the value of while loop operator, k=k+1.23:
**end while**




**Figure 6:**
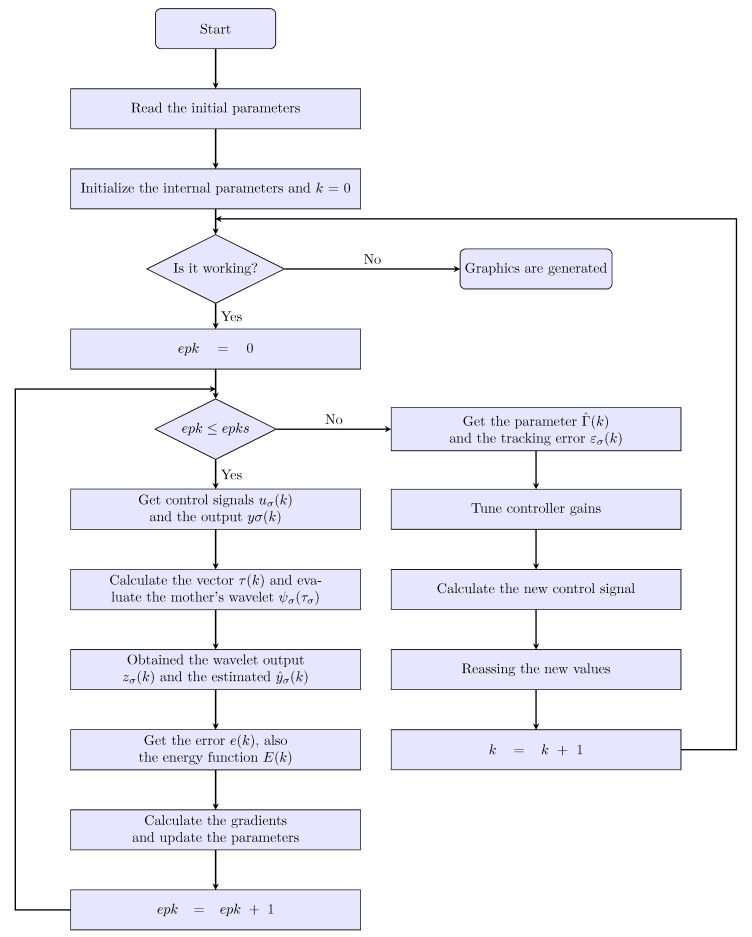
Flowchart of the PID wavenet algorithm proposed.

The flowchart for the PID wavenet algorithm is illustrated in Figure 6.

## 4. The Experimental System

The goal of this section is to present the experimental platform as well as the design of experiments.

### 4.1. Experimental Platform

**Figure 7:**
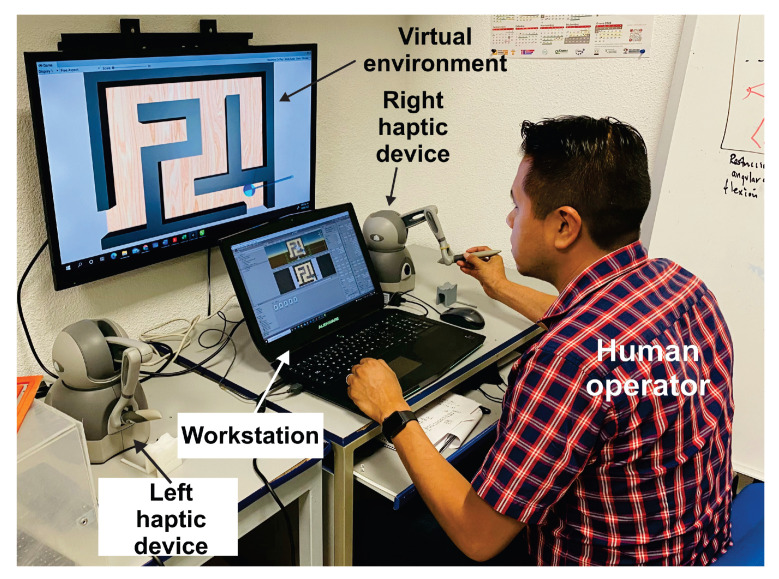
Experimental platform shows the user solving a virtual maze with a his right hand, which commands a (right) haptic interface.

Consider a *Geomagic Touch* [38], as the haptic interface for each hand, modeled as a three degrees of freedom nonlinear robot given in (3) and (4), see Figure 7. A PC equipped with an Intel(R) Core(TM) i7-4720HQ CPU runs at 2.60 GHz, 16 GB RAM and graphic card NVIDIA GeForce GTX 980M, under OS Windows 10, and Unity3D, release 22 March 2020. System deploys a soft real-time thread that updates the whole haptic control loop at [*h* = 1 ms], corresponding a fast enough sampling frequency of 1KHz for the kinesthetic stimulus, while visual renderization is update at 66 Hz.

### 4.2. Design of Experiments

The experiments aim at evaluating competency of solving a maze with motor commands within a given order and precision, involving executive decision making and motor patters, using PMT protocol. It is surmised that such motion patters leads to coordinate bimanual cooperation of both hands that improves under haptic guidance. Then, it is considered two experiments, one providing only haptic stimuli when user touches la boundaries of the maze and other one with continuous haptic guidance, not only when it deviates as much as touching the limits of the maze.

**Figure 8:**
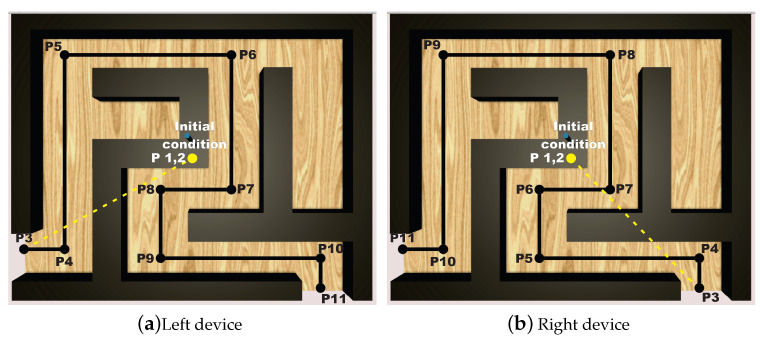
Multiple branch maze, showing the left-hand (**a**) and right-hand (**b**) solution for SCM excercise.

To this end, the user solves the maze by commanding the 3D haptic interface, which is represented in the virtual world as the pointer within the virtualized maze as shown in Figure 8. The middle road of the maze is considered as the position reference, then the task of the haptic control is to converge to such position reference path, whatever how the user navigates to solve the maze. In this way, the novel paradigm of invariant motor learning is implemented in our scheme: User tracks at his/her own pace and motor capacity the defined invariant position points $P_{i}$ shown in Figure 8, i.e., the algorithm does not impose a desired time base since desired velocity is neither enforced visually nor imposed throughout the control scheme. In this way, user intentional movement is deployed to solve the maze at will, which es essential for motor rehabilitation.

Two exercises are designed, depending of two level of difficulty are instrumented by considering: (a) Low difficulty represented by a Simple Connection Maze (SCM), where a unique non-branching path solves the maze, and (b) Medium difficulty represented by Multiple Connection Maze (MCM), where there exists multiple branches and dead-ends requiring executive decision making to transverse until reaching the exit.

### 4.3. How the Haptic Control Occurs and How Human Is Guided Spatially

**Figure 9:**
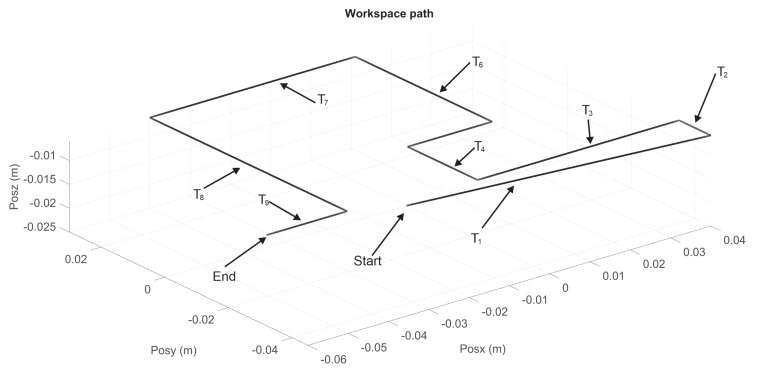
Transects $T_{i}$ trajectories for right haptic device for MCM test.

**Figure 10:**
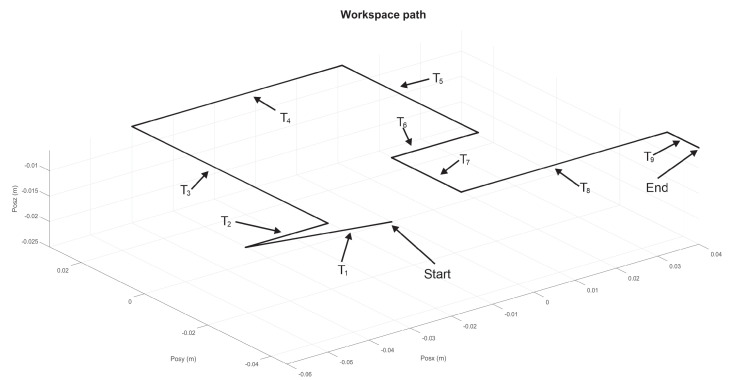
Composite task from $T_{i}$ trajectories for left haptic device for MCM test.

Let Figure 9 and Figure 10 show the nominal trajectory for the left and right hand SCM and MCM, where $T_{i}$ represents the nominal transect segments. The ends of each $T_{i}$ are constant spatial points. Assuming that haptic device pointer is at any given instant in a given spatial Cartesian location $\xi_{r}$, the closest $T_{i}$ is chosen, and it is determined the closest point $\xi \in T_{i}$ as the reference point at that instant, i.e., $y_{\sigma -ref}=\xi_{r}$. Then, the controller injects a torque $u_{\sigma }Nm$ to the haptic device to attract $\xi_{r}\to y_{\sigma -ref}$. In this way, wherever the pointer is, it is attracted to the closest point of transect $T_{i}$, independently of time, and independently of how fast or slow the velocity of user pointer is. Since human fingertip is inserted at thimble of the haptic device, then human perceive such torque as a vector of haptic force $f_{h}$, given by $f_{h}=J{\left(q\right)}^{-1}u_{\sigma }N$.

**Figure 11:**
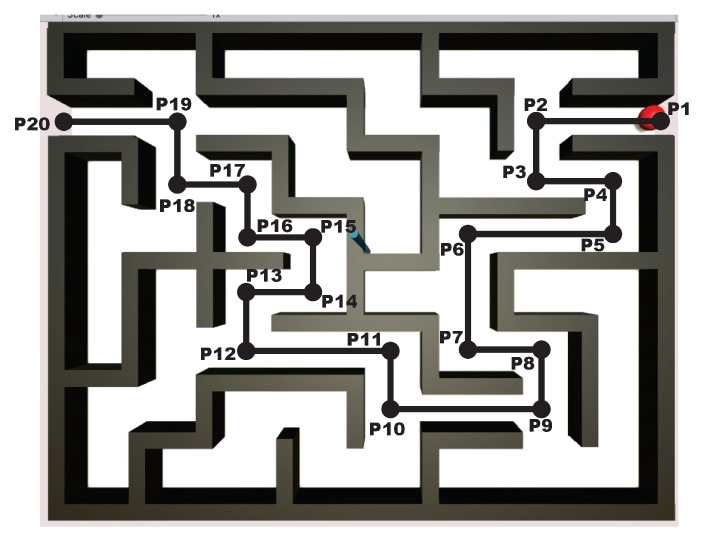
Solution to the maze MCM of first exercise.

**Figure 12:**
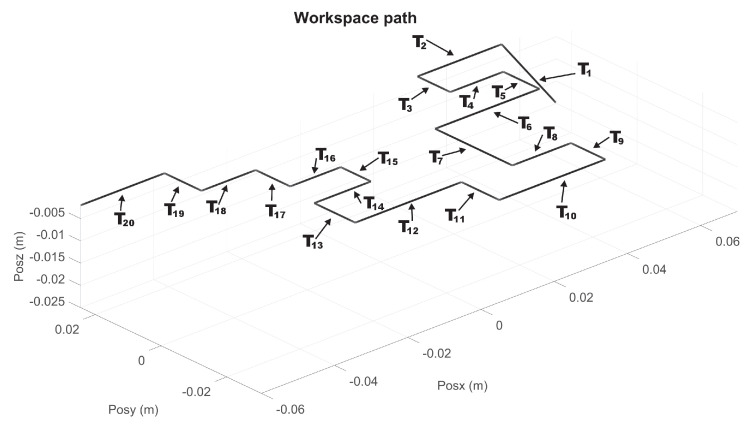
Composite task from $T_{i}$ transects for right haptic device of exercise 2 (MCM).

**Figure 13:**
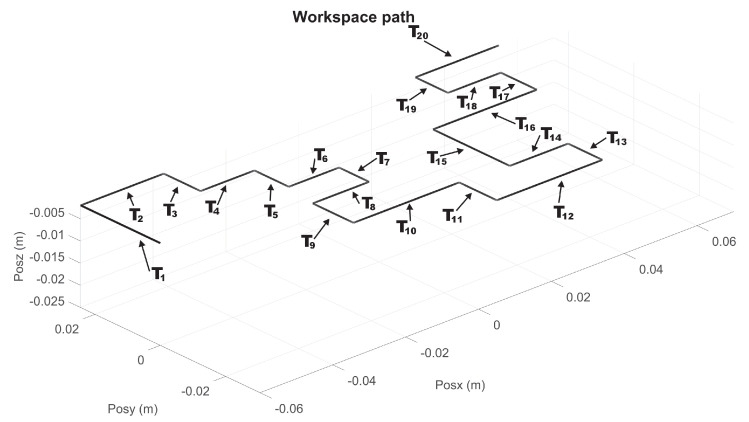
Composite task from $T_{i}$ transects for left haptic device of exercise 2 (MCM).

For the MCM exercises, a maze of medium difficulty is shown in Figure 11, whose virtualization was programmed in Unity3D, with a unique solution for both right and left haptic devices. Now, 20 via points are considered, for i=19 transects $T_{i}$, starting at P1, see Figure 12 and Figure 13 for right and left haptic devices, respectively.

## 5. Experimental Results

A pre-training phase is performance to obtain the initial values of the neural network parameters, see Table 1 and Table 2. THis phase is conducted in a human-in-the-loop configuration.

### 5.1. Experiment 1: Active Haptic Guidance with SCM and MCM

**Figure 14:**
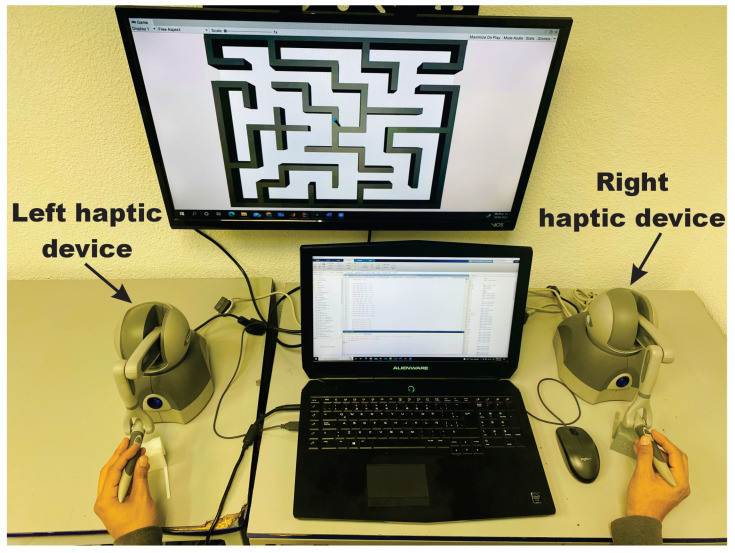
Initial operating position by the user.

**Figure 15:**
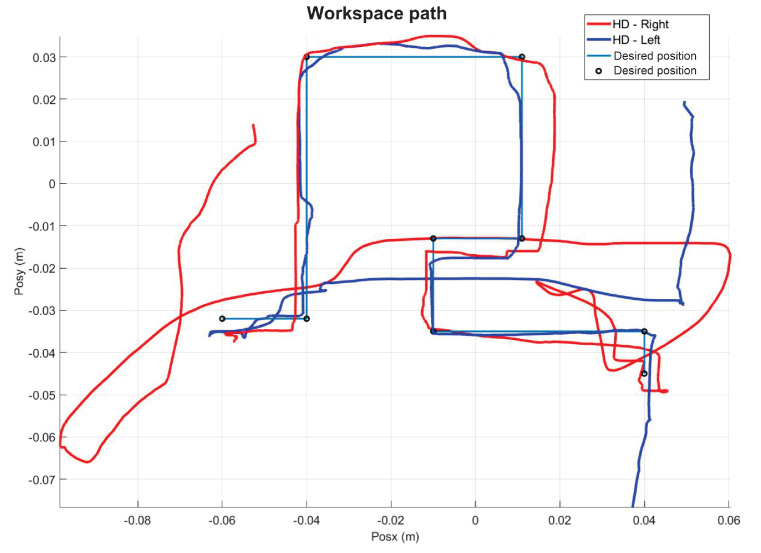
Workspace trajectory of two haptic devices in an active haptic guidance task for SCM.

**Figure 16:**
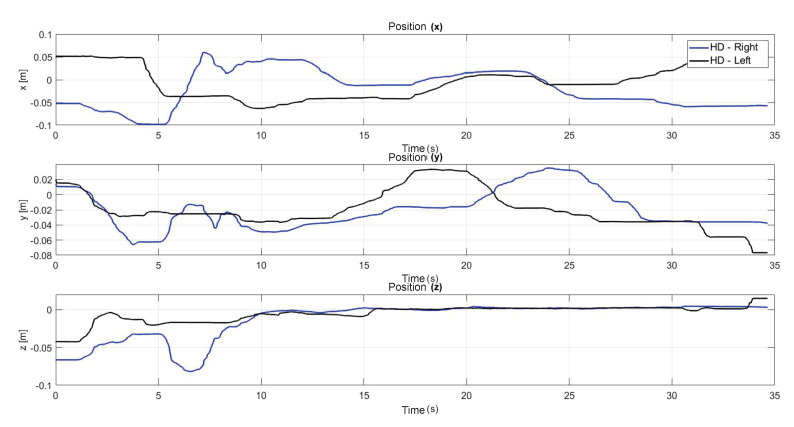
Operational position of two haptic devices in active haptic guidance task for SCM.

Figure 14 shows the initial position that the user must have when starting each of the experiments. Figure 15 shows the virtual navigation behavior of the user in the workspace to solve the SCM bimanually, with smooth position behaviour, as shown in Figure 16.

In this passive navigation configuration, user exhibits low performance since haptic guidance not only is scarce but intermitent (user perceives a force at fingertip only when it touches the walls of the maze).

### 5.2. Experiment 2: Passive Haptic Guidance with SCM and MCM

**Figure 17:**
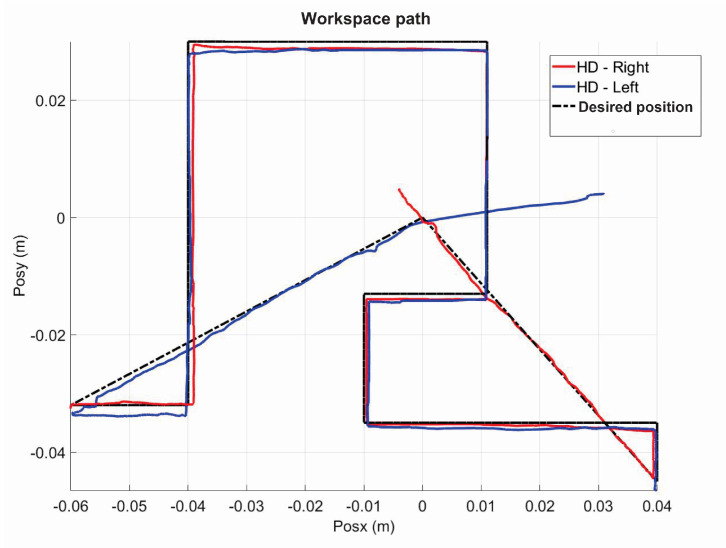
Workspace trajectory of two haptic devices in passive haptic guidance task: exercise 1.

**Figure 18:**
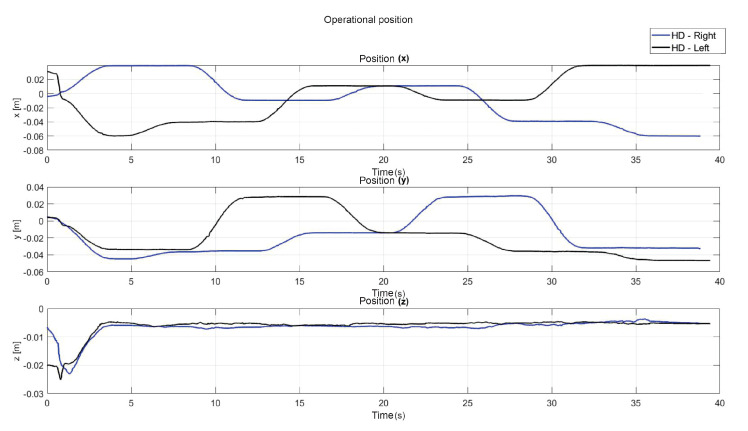
Operational position of two haptic devices in passive haptic guidance task: exercise 1.

**Figure 19:**
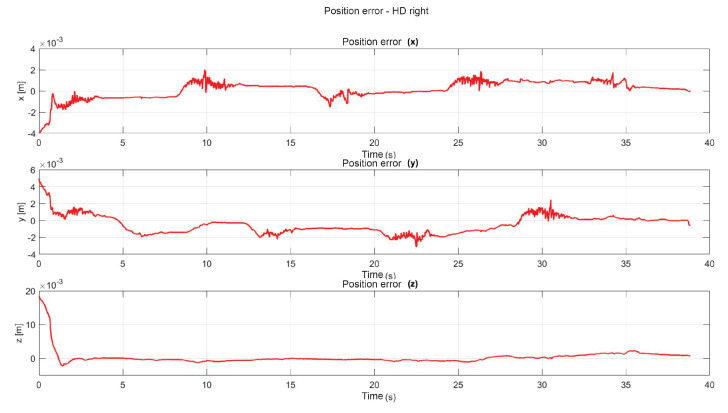
Right haptic device position error: exercise 1.

**Figure 20:**
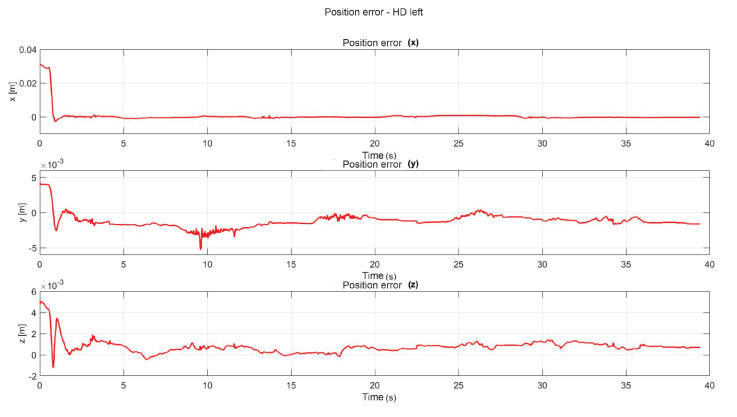
Left haptic device position error: exercise 1.

**Figure 21:**
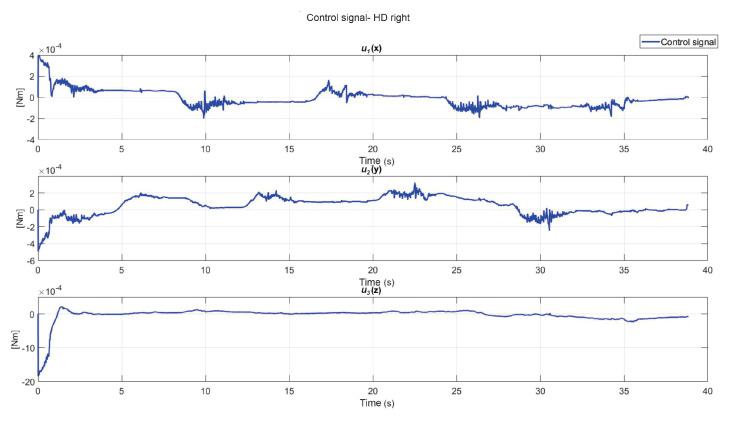
Right haptic device control signal: exercise 1.

**Figure 22:**
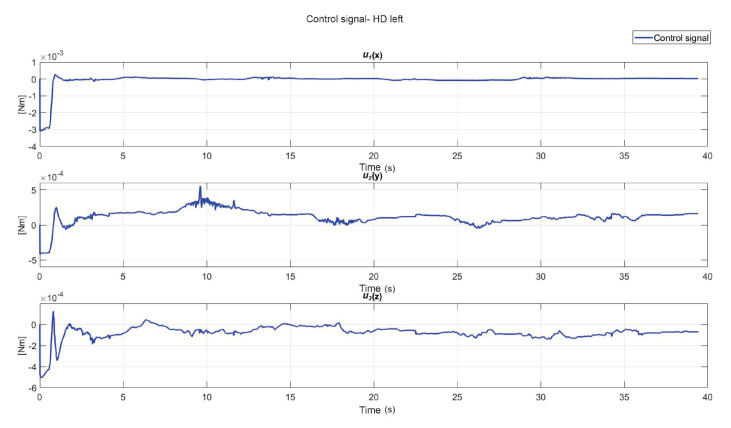
Left haptic device control signal: exercise 1.

The following exercise consists of the implementation and application of a control law for trajectory tracking, from the construction of a desired trajectory through motion planning (Figure 17), a different one for each of the haptic devices integrated in the platform (Figure 18). The experiment consists of each device performing tracking-based regulation with the user in the loop, giving the user visual and force feedback on the planned trajectory, where the applied controller guarantees position convergence, the goal is that all this can be used for rehabilitation purposes. After the development of exercise 1, Figure 19 and Figure 20 show the position errors of each of the haptic devices. Figure 21 and Figure 22 show the control signal that is sent to each device to generate trajectory tracking.

### 5.3. Exercise 1: Passive Haptic Guidance without User in the Loop

**Figure 23:**
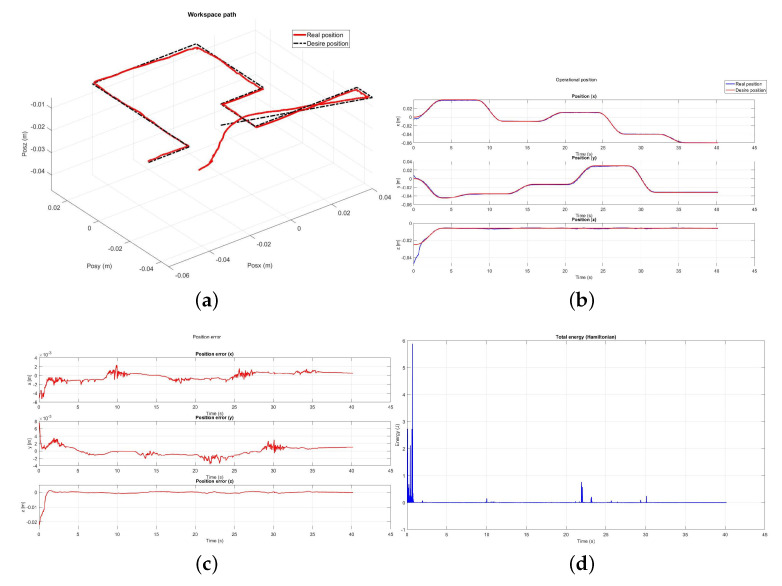
Experimental results with user in the loop: (**a**) Workspace path, (**b**) Operational position, (**c**) Position error, (**d**) Total energy in the task.

For the next test, passive haptic guidance is applied on the device without user in the loop, Figure 23 shows the results in position convergence and energy exchange.

### 5.4. Exercise 1: Passive Haptic Guidance with User in the Loop and Disturbances

**Figure 24:**
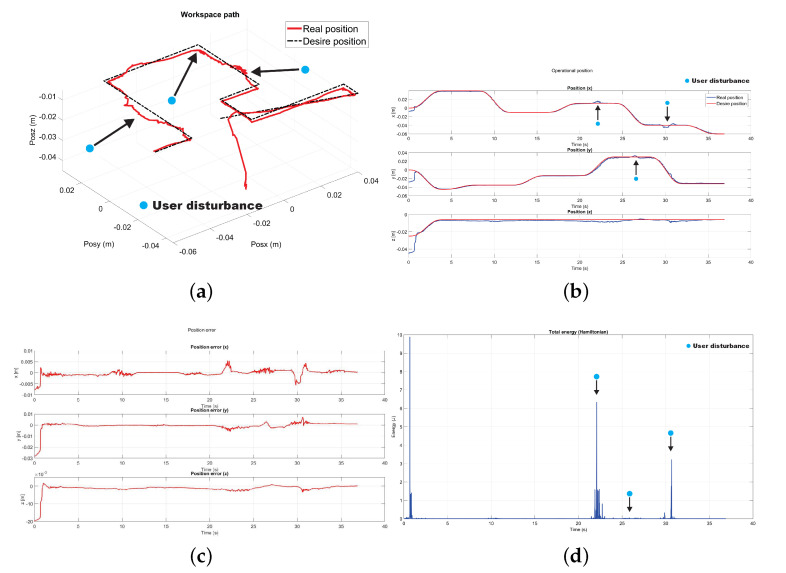
Experimental results with user in the loop: (**a**) Workspace path, (**b**) Operational position, (**c**) Position error, (**d**) Total energy in the task.

The following test was performed to check the effectiveness of the controller implemented to compensate uncertainty and disturbance generated by the user when it is coupled with the device. Figure 24 shows the moments where there are disturbances, the same instant where there is an increase of energy, the same that the controller uses to compensate and redirect the device to the desired trajectory.

### 5.5. Exercise 2: Active Haptic Guidance

**Figure 25:**
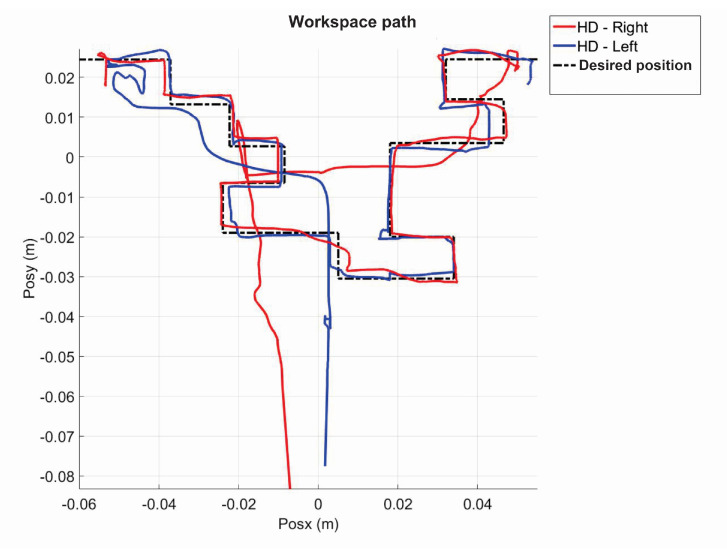
Workspace trajectory of two haptic devices in active haptic guidance task: exercise 2.

**Figure 26:**
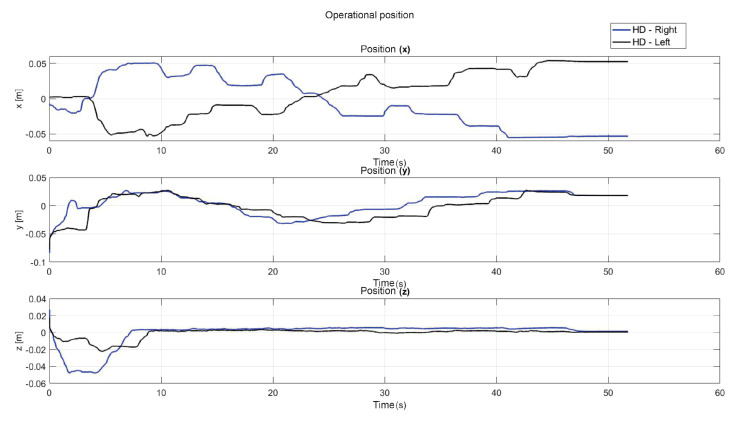
Operational position of two haptic devices in active haptic guidance task: exercise 2.

The present subsection shows the experimental results for the case of Active Haptic Guidance. The Figure 25 shows the behavior of the two haptic devices in the workspace and Figure 26 shows the operational position of two haptic devices in active haptic guidance task.

### 5.6. Exercise 3: Passive Haptic Guidance

The platform with two haptic devices (right hand and left hand), was evaluated in 2 different experiments (mazes with different level of difficulty), each maze with 2 conditions (without control and with control), as defined below: (i) The user uses two haptic devices to solve a maze in free motion (active haptic guidance), i.e., the user controls their own movements. In this condition (without interactive forces), the execution time is allusive to the performance of each user (different in each hand); visual feedback plays an important role. The compensation of the vector of gravitational forces is established. The optical encoders of the haptic device allow performance measurement by mapping the vector of joint variables to the operational space; and (ii) The user interacts with the platform actively, that is, a tracking control law is implemented, which has the objective of teaching the user how to solve the maze. The control law has the goal of compensating for the uncertainties generated by the user when performing the task (disturbances and position errors). In these conditions (adaptive force that conditions the guide in the operational space), it describes a kinesthetic learning task. The performance of each user in the task represents involuntary movements of the trajectory and establishes the energy requirement, defined in an adaptive way by the control.

**Figure 27:**
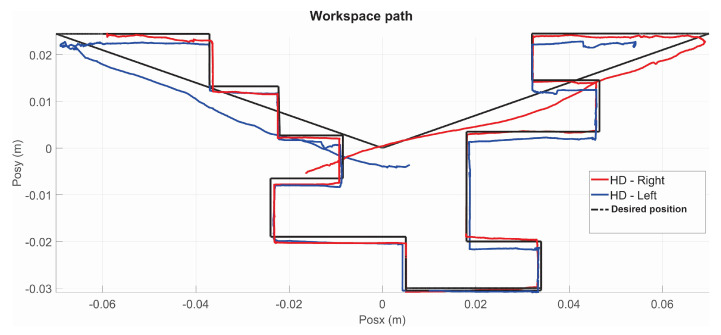
Workspace trajectory of two haptic devices in passive haptic guidance task: exercise 3.

**Figure 28:**
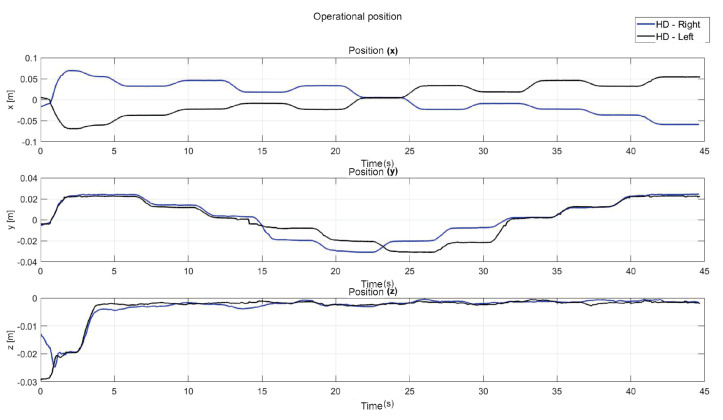
Operational position of two haptic devices in passive haptic guidance task: exercise 3.

**Figure 29:**
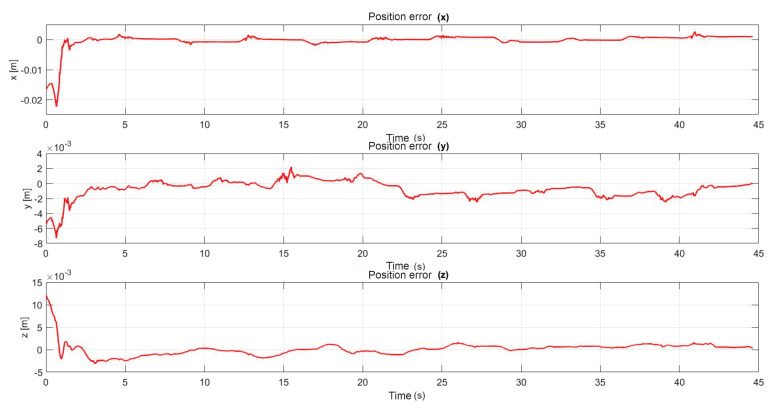
Position error of right haptic device: exercise 3.

**Figure 30:**
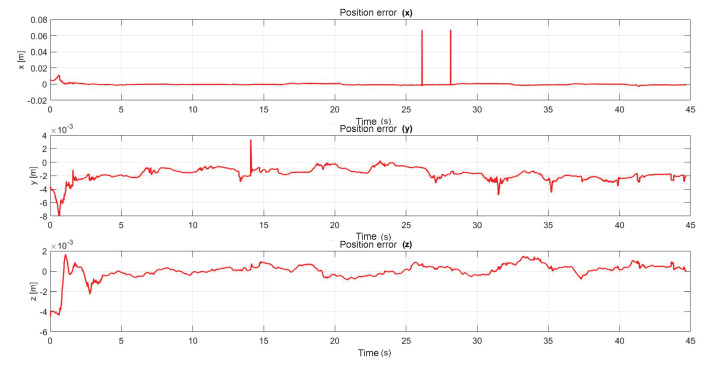
Position error of left haptic device: exercise 3.

As a result of the application of the exercise on the labyrinth of medium difficulty, Figure 27 is presented, which corresponds to the trajectory on the workspace of the two haptic devices, in Figure 28 the position operation on each axis of both devices (x, y, z), Figure 29 and Figure 30 show the position errors generated from tracking the desired trajectory of the two haptic devices. These graphs show the performance of the controller in passive haptic guidance tasks in position tracking and convergence.

### 5.7. PID Wavenet-IIR Parematers

**Figure 31:**
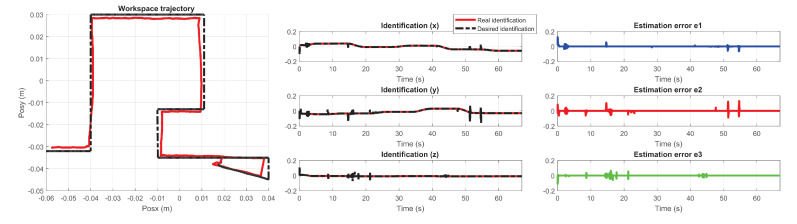
Performance PID wavenet-IIR controller for exercise 1. In this case, the robot was used for σ=r (right robot).

**Figure 32:**
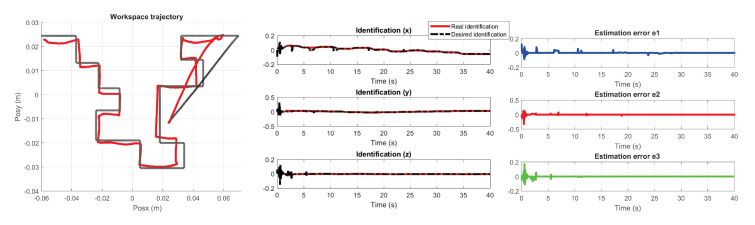
Performance PID wavenet-IIR controller for exercise 2, σ=r.

The performance of the PID wavenet control was evaluated based on the convergence time to the desired trajectory. The following figures describe the behavior of the adaptive control implemented on the maze of exercise 1 and 2. Figure 31 and Figure 32 show the trajectory tracking in the workspace, the response estimation of the plant (haptic device), as well as the maze estimation error of exercise 1 and exercise 2 respectively.

**Figure 33:**
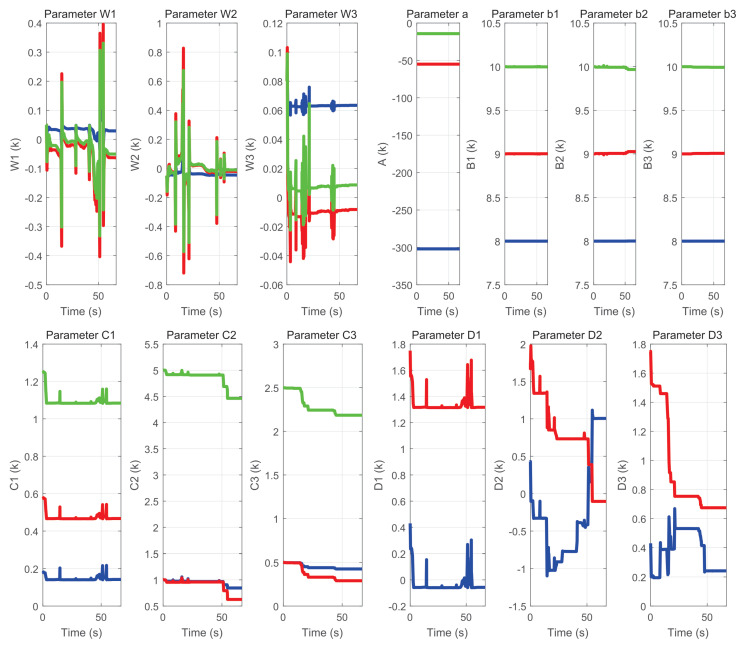
Behavior of neural network parameters used in the labyrinth of exercise 1.

**Figure 34:**
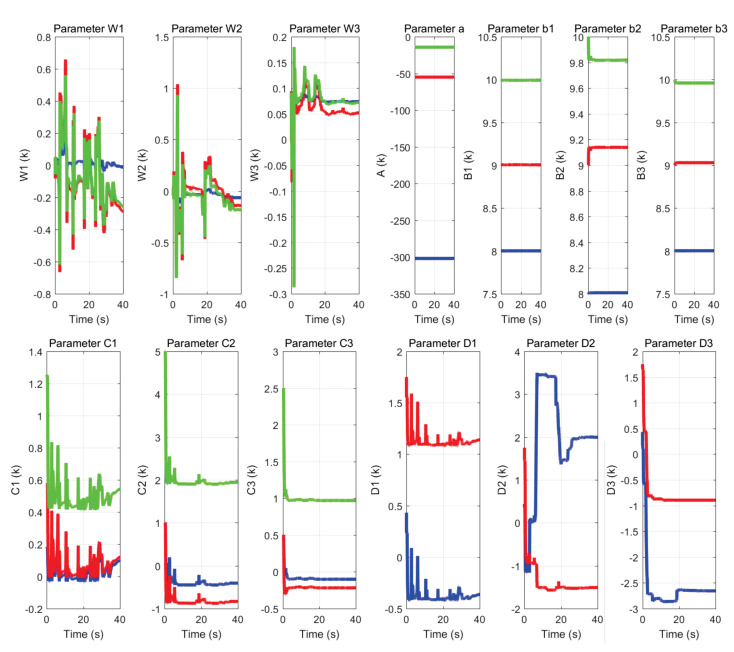
Behavior of neural network parameters used in the labyrinth of exercise 2.

Figure 33 and Figure 34 show: (a) the neural network weights *W*, (b) parameters *a* and *b*, where *a* is the scaling variable, which allows for dilations and contractions; and *b* is the translation variable, which allows for displacement at instant *k*, as well as (c) parameters *C* and *D* which are the forward and backward coefficients of the IIR filter respectively.

It is observed that all of them change their value in each instant of time of the exercise, as they evolve to the dynamics generated by the user and the region in which the haptic device is located within the workspace.

**Figure 35:**
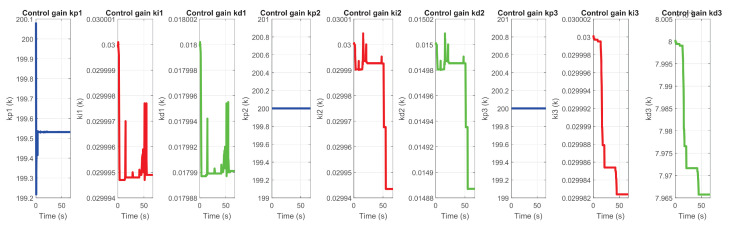
Self-tuning of gains $k_{p}$, $k_{d}$ and $k_{i}$ in exercise 1.

**Figure 36:**
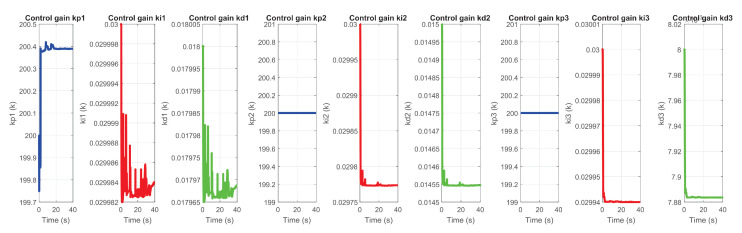
Self-tuning of gains $k_{p}$, $k_{d}$ and $k_{i}$ in exercise 2.

Figure 35 and Figure 36 corresponds to the behavior of the PID gains, auto-tuned online for each degree of freedom of the device.

## 6. Conclusions and Future Work

### 6.1. Conclusions

A novel identification and control scheme for the 3D nonlinear haptic robotic devices is implemented efficiently based in wavenet with IIR filter; it identifies inverse dynamics aimed at tuning PID feedback gains, not to approximate dynamics as usual neural networks-based control. Purposely, this scheme yields self-tuning of feedback gains to react to human interaction and commanding forces, notably, without any a priori knowledge of the haptic device to guarantee global asymptotic convergence. Real-time human-in-the-loop bimanual experiments show human cooperative decision making since both hands maneuver in the same workspace. The proposed scheme is viable for for practical implementation, where typically not only the exact nonlinear dynamics is now known but it accounts to varying and persistent exciting human interacting force. There was implemented the patterns of a clinical test with a healthy volunteer to assess the usefulness of the platform in real conditions, showing potential for patients.

### 6.2. Future Work

Platform was tested with healthy subject exhibiting velocities and range of motion within the expected regimes of patients. Next step is to run a controlled and clinically supervised protocol with upper limb motor disability patients who require motor rehabilitation. Particular interest is on cerebrovascular accident patients that requires also motor and cognitive coordination, for which virtual mazes tests are an option.

## Figures and Tables

**Table 1:** Proposed values for wavenet-IIR PID controller.

Parameter	Value	Parameter	Value	Parameter	Value
Neurons, *L*	3	$\mu_{W_{\sigma }}$	0.5	$\mu_{p}$	[0.002,0.002,0.002]
Feed-back, *M*	3	$\mu_{a_{\sigma }}$	0.5	$\mu_{i}$	[0.002,0.002,0.002]
Feed-forward, *N*	2	$\mu_{b_{\sigma }}$	0.5	$\mu_{d}$	[0.004,0.004,0.004]
Epocs, epk	50	$\mu_{C_{\sigma }}$	0.5	$K_{p_{\sigma }}\left(k\right)$	[200,200,200]
	$\mu_{D_{\sigma }}$	0.5	$k_{i_{\sigma }}\left(k\right)$	[0.018,0.015,0.008]	
	$v_{\sigma }\left(k\right)$	0.5	$k_{d_{\sigma }}\left(k\right)$	[0.3,0.03,0.03]	

**Table 2:** Initial values of the parameters of wavenet and PID controller.

Parameter	Value
$W_{\sigma }$	$\left[\begin{array}{ccc} 0.05 & 0.05 & 0.05\\ -0.05 & -0.05 & -0.05\\ 0.08 & 0.08 & 0.08 \end{array}\right]$
$a_{\sigma }$	$\left[\begin{array}{ccc} -302 & -55 & -14.2 \end{array}\right]$
$b_{\sigma }$	$\left[\begin{array}{ccc} 8 & 9 & 10\\ 8 & 9 & 10\\ 8 & 9 & 10 \end{array}\right]$
$C_{\sigma }$	$\left[\begin{array}{ccc} 0.18 & 0.576 & 1.25\\ 1 & 1 & 5\\ 0.5 & 0.5 & 2.5 \end{array}\right]$
$\mu_{C}$	0.5
$D_{\sigma }$	$\left[\begin{array}{cc} 0.43 & 1.75\\ 0.43 & 1.75\\ 0.43 & 1.75 \end{array}\right]$

## Data Availability

All the data recorded during the tasks of active haptic guidance and passive haptic guidance on the human-robot interaction platform can be provided by the authors of this paper upon request.
